# The effect of angular dispersion on THz data transmission

**DOI:** 10.1038/s41598-022-15191-w

**Published:** 2022-06-29

**Authors:** Rabi Shrestha, Zhaoji Fang, Hichem Guerboukha, Priyangshu Sen, Goretti G. Hernandez-Cardoso, Enrique Castro-Camus, Josep M. Jornet, Daniel M. Mittleman

**Affiliations:** 1 School of Engineering, Brown University, Providence, RI 02912 USA; 2 Department of Electrical and Computer Engineering, Northeastern University, 360 Huntington Ave, Boston, MA 02115 USA; 3 Department of Physics, Philipps-Universität Marburg, Renthof 5, 35032 Marburg, Germany

**Keywords:** Engineering, Electrical and electronic engineering

## Abstract

One of the key distinctions between legacy low-frequency wireless systems and future THz wireless transmissions is that THz links will require high directionality, to overcome the large free-space path loss. Because of this directionality, optical phenomena become increasingly important as design considerations. A key example lies in the strong dependence of angular radiation patterns on the transmission frequency, which is manifested in many different situations including common diffraction patterns and the emission from leaky-wave apertures. As a result of this effect, the spectral bandwidth at a receiver is nonlinearly dependent on the receiver’s angular position and distance from the transmitter. In this work, we explore the implications of this type of effect by incorporating either a diffraction grating or a leaky wave antenna into a communication link. These general considerations will have significant implications for the robustness of data transmissions at high frequencies.

## Introduction

As the demand for higher data rates increases, modern communication systems will eventually face a bottleneck. Many experts now speculate that terahertz (THz) wireless communication systems can be a solution this problem^1^. THz links operate at higher carrier frequencies (above 0.1 THz) than current 5G systems and can potentially support much larger bandwidths^2,3^. At THz frequencies the channel faces increased path loss, increasing in proportion to ${\nu }^{2}$, and will therefore require high-gain antennas to compensate for this loss. Therefore, in such systems THz beams are likely to be highly directive. This directivity raises a number of interesting challenges in the design and implementation of THz systems. For one, these directional beams will need to be steered in order to maintain a link with a mobile receiver^4,5^. The directionality also requires new protocols for the initial discovery and establishment of links and for overcoming transient blockage of the beam by, e.g., mobile obstructions such as people, and also has important implications for link security^6^. Numerous innovative solutions to these problems have been proposed recently, and these issues remain topics of much current research^7–15^.

Because of the high directionality, one can make some very general statements about the properties of propagating beams. Here, we focus on one point, which is that directional beams can readily manifest phenomena associated with diffraction^16^. This is of course well known in the context of optics^17,18^, but some of the consequences have received less attention in the RF and wireless communication communities^19^. One important consequence of diffraction is that far-field radiation patterns can depend very strongly on frequency. The central idea is that, as a broadband signal propagates from a transmitter to a receiver in the far field, different spectral components within the signal’s bandwidth will propagate at slightly different angles, resulting in a spreading of the information content across angle. The quality of the received signal therefore depends in a complicated way on the size of the receiver’s aperture, compared to the degree of angular spreading (which in turn depends on the broadcast bandwidth). We suggest that these effects will be very difficult to avoid; indeed, in most of the proposed ideas referenced above, one can expect that strong frequency-dependent beam patterns will be imposed on transmitted signals (either intentionally or unintentionally). The implications for link quality can be quite significant. We note that, at low frequencies typically used for legacy communication networks, these effects are to some extent masked by both multi-path interference and by the wide quasi-omnidirectional nature of broadcasts, and so beam diffraction effects are generally not an engineering consideration for network designers. However, at higher frequencies where multi-path effects are suppressed and beams are highly directional, the resulting phenomena are prominent and can in many cases be deterministically predicted. Thus, their deleterious impact on the quality of data transfer can be isolated from other effects such as multi-path fading, and in some cases possibly even corrected. This is quite distinct from the situation encountered at lower frequencies.

In this paper, we explore these phenomena by introducing several different quasi-optical elements into a THz wireless link, and characterizing their impacts on link quality. Our approach is to add strong angular dispersion to a wireless link, and explore the consequences for the quality of data transfer (as quantified by the bit error rate of a transmission). In the realm of optics, the prototype device for introducing angular dispersion is a diffraction grating, for which the propagation angle of an emerging beam is directly proportional to the free-space wavelength. Although it is not common to find a passive diffraction grating used in a wireless system^20^, it is nevertheless a useful starting point for our discussion about the impact of angular dispersion on link quality. Subsequently, we consider a leaky-wave antenna (LWA), which also exhibits a large angular dispersion. Indeed, in recent work by us and others, this angular dispersion has been directly exploited for valuable network functions^15,21–26^. These earlier studies have so far not considered the implications on link quality of employing strong angular dispersion for broadband data transmissions. We show that a transmitter and receiver attempting to establish a direct line of sight (LOS) wireless link with strong angular dispersion can encounter additional loss beyond the conventional free-space path loss, which can vary nonlinearly with receiver angle. If not corrected, this phenomenon can have profound implications for many aspects of system operation.

Conventionally the Friis equation, can be used to estimate the received power of a signal radiated from a transmitter, according to

1 $${P}_{rx}\left(dB\right)={P}_{tx}+{G}_{tx}+{G}_{rx}+20{\mathrm{log}}_{10}\frac{\lambda }{4\pi R}$$

where $\lambda$ is the free-space wavelength, ${P}_{tx}$ is the transmitted power, ${G}_{rx}$ is the gain of the receiver antenna, ${G}_{tx}$ is the gain of the transmitter antenna and $R$ is the distance of the link. In the simplest case of an isotropic radiator, for a given wavelength the attenuation of the signal is 20 dB per decade (20 dB added loss for every tenfold increase in distance). When an antenna or other element introduces angular dispersion in the link, the attenuation increases because the radiation angular distribution spreads due to the fact that different frequencies propagate in different directions. For a given angular dispersion, this effect is compounded as the distance between the transmitter and receiver is extended because the spectrum of the transmitted signal is spread over a larger range in space, such that the (presumably fixed) receiving aperture acts as a spectral filter, detecting only a portion of the original bandwidth. As a result, the bit error rate for a received signal can drop more rapidly than would be predicted by the Friis equation (in other words, faster than the drop corresponding to 20 dB signal to noise ratio (SNR) decrease per decade of range). Moreover, this filtering is dependent on both angle and distance from the diffracting aperture. In this work, we first characterize the increased attenuation resulting from the angular dispersion of a diffraction grating. Then, we show that using a LWA for transmitting THz waves also results in a similar angle-dependent attenuation. We transmit multiple frequency components over a broad bandwidth, to illustrate a scenario in which frequency division multiplexing (FDM) is employed. Finally, we send data using our LWA and we show the impact of angle-dependent attenuation on the received SNR.

### Diffraction grating

As a first step to demonstrate the effects of diffraction, we employ a variable reflective diffraction grating, which has been developed for reflective beam-steering^27^. A THz beam incident normal to a diffraction grating produces a series of reflected beams, the so-called diffraction orders. For a given grating period, the angle of the $m$th order can be found from:

2 $$\mathit{sin}\theta =\frac{mc}{\Lambda \nu }$$

where $\Lambda$ is the grating period, $\nu$ is the carrier frequency (200 GHz in our experiments) and $c$ is the speed of light. In this work, we focus only on the first order mode so $m=1$. If the carrier signal is modulated with data, as is the case in all communication systems, the different components of the broadband spectrum will spread in angle accordingly. The spectral bandwidth of the signal received by a receiver located in the far field at an angle θ, with an aperture of angular size $\Delta \theta$, can be calculated by taking the derivative of Eq. (2). We assume that the distance of the receiver, $R$, is far greater than the aperture of the receiving antenna, ${A}_{apt}$. This allows us to make the approximation for the acceptance angle as $\Delta \theta \approx {A}_{apt}/R$, resulting in

3 $$\Delta \nu =\frac{c}{\Lambda sin\theta tan\theta }\Delta \theta$$

If the bandwidth of the transmitted signal exceeds the value predicted by this result, then the detected signal’s bandwidth is filtered by the receiver aperture Δθ, and is given by Eq. (3) rather than by the transmitted value. In this case, the received spectral bandwidth is inversely proportional to the radial distance and depends nonlinearly on the receiver position, $\theta$. This conclusion demonstrates an important implication of using any diffractive device for beam-steering a modulated signal: the spectral bandwidth will become limited and the signal quality will be reduced as the angle and distance increases.

**Figure 1:**
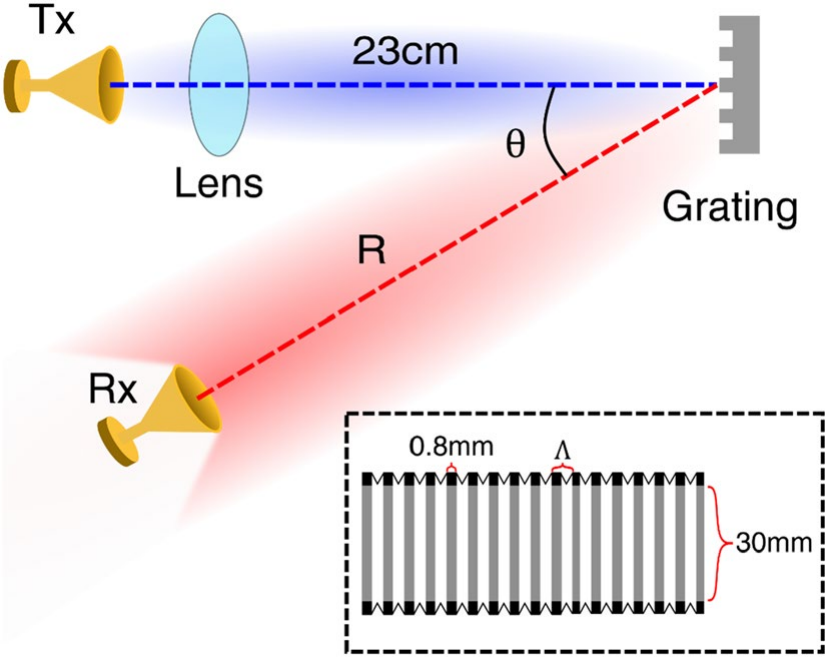
Experimental setup of diffraction grating measurement. The transmitter radiates a beam normal onto the diffraction grating and the beam is reflected at angle $\uptheta$ based on the periodic spacing $\Lambda$. The inset shows a close up of the reflective gratings ‘accordion’ design.

In order to demonstrate the impact of the decrease in spectral bandwidth on a wireless communication link, we employ the setup shown in Fig. 1. A THz wave is generated by a frequency multiplier chain (FMC) generating a signal with carrier frequency of 200 GHz, and with an on–off keying (OOK) modulation of 1.12 Gbps, coupled to a conical horn antenna and dielectric lens, for a net transmitter antenna gain of 34 dBi. This vertically polarized modulated wave is directed towards the diffraction grating (also vertically oriented). The first-order diffracted signal then spreads in space. At the receiver’s position, only a portion of the transmitted bandwidth is detected. We use a zero-bias Schottky diode (ZBD) as the detector, also coupled via a conical horn antenna (receiver gain = 21 dBi). Our variable diffraction grating permits us to vary the grating spacing $\Lambda$ mechanically from 1.2 to 2.6 mm, thereby tuning the received bandwidth as predicted by Eq. (3). For illustrative purposes, we characterize the radiation pattern for a grating spacing of 2.58 mm, which result in a primary lobe angle for the first-order diffracted beam of 35.5° (according to Eq. (2)).

**Figure 2:**
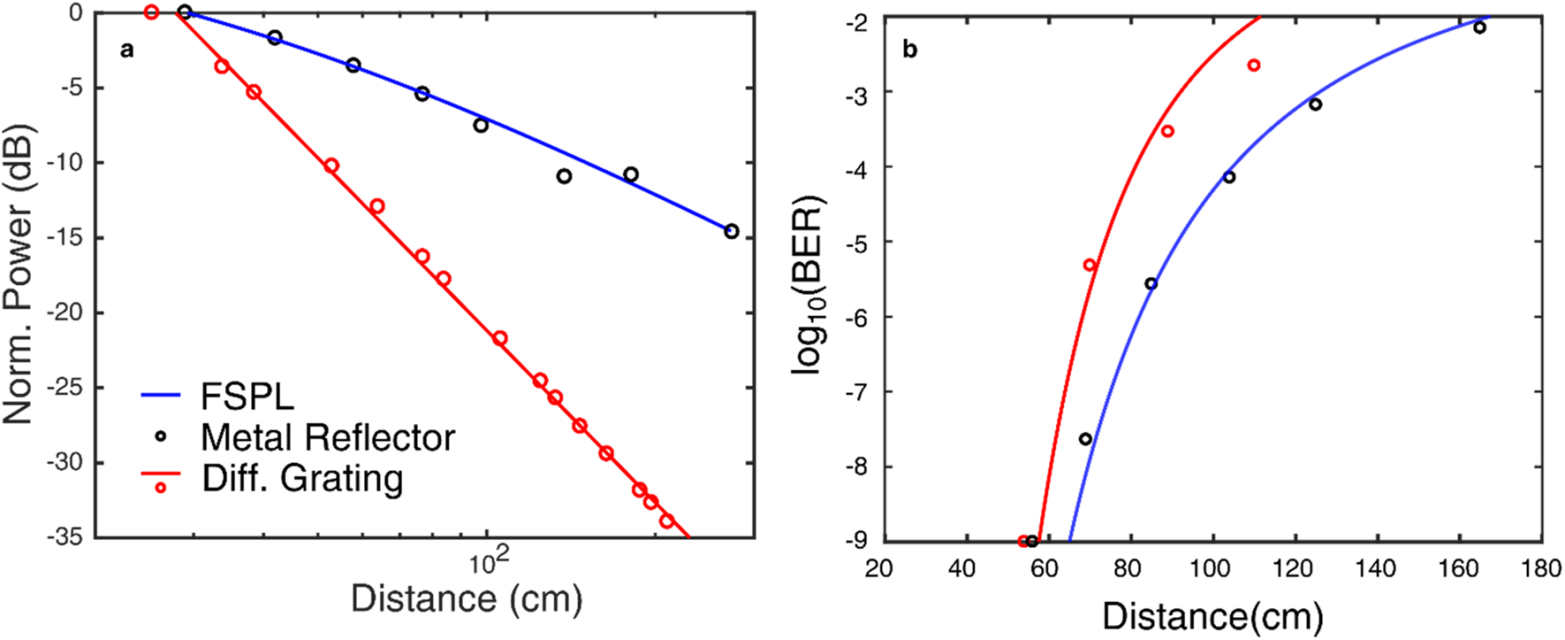
(**a**) The normalized power detected at the receiver positioned at an angle of 35.5° for a metal reflector (black circle), and angles corresponding to the primary lobes of the diffracted signal when the grating spacing is $\Lambda =2.58\mathrm{ mm}$ (red circle). As the distance increases the attenuation of the signal is greater when using a diffraction grating than compared to a metallic reflector. The slope of the linear fit is 34.7 (dB/decade) for the signal diffracted by the grating (red line). The FSPL is also plot against the metal reflector data (blue line). (**b**) The measured BER for the uncompressed grating (red dots) is greater compared to a metal reflector (black dots) as the receiver distance is increased for a given transmitter power. The calculated BER for the metal reflector (blue line) only experiences FSPL and increases at a slower rate in comparision to the higher BER predicted for the signal from the diffraction grating (red line).

The results for the detected power and bit error rate (BER) as the receiver distance is increased are shown in Fig. 2. We compare these results to those obtained when the grating is replaced by a flat metal reflector (i.e., a mirror), which introduces no angular dispersion, so that one expects the customary 20 dB per decade free-space path loss. Compared to the flat reflector, the signal from the diffraction grating exhibits an attenuation of 34.7 dB per decade, notably larger than free-space path loss. This result can be understood by the analytical expression in Eq. (3). As the grating is compressed (decreased grating period), the spectral bandwidth is decreased, leading to a further increase in the loss. This clipping of the spectrum is also manifested in differences in the measured BER (Fig. 2b). The consequence of increased attenuation from angular dispersion leads to a higher rate of increase in the BER at greater link distances, compared to the BER change due to FSPL.

### Leaky wave antenna

Next, we show the effects of transmitting data using dispersive antennas such as a LWA. This idea is motivated by the numerous recent studies in which LWA architectures have been targeted as valuable for broadband THz communication systems. Several of these ideas involve exploiting the LWA’s angular dispersion properties for sensing tasks necessary for network operation, although their use for data transmission (e.g., multiplexing) has also been explored recently. To explore the implications of transmitting data using an antenna with such strong angular dispersion, we employ a metallic parallel plate waveguide (PPWG), separated by an air core, with a rectangular slot (with a width of 1 mm and a length of 30 mm) in one of the plates to allow a portion of the guided wave to leak into free space. This configuration serves as a prototype for any leaky-wave device manifesting a strong (and nonlinear) coupling between frequency and emission angle. In this study, we select a plate spacing of *b* = 1.60 mm, which defines a single-mode operation band for the waveguide between the lowest (TE_1_ mode) cutoff frequency at 90 GHz and the second-order TE mode cutoff at 180 GHz. As in the case of the diffraction grating, the transmitted beam exhibits a nonlinear relation between frequency and angle.

4 $$\nu =\frac{c}{2bsin\theta }$$

**Figure 3:**
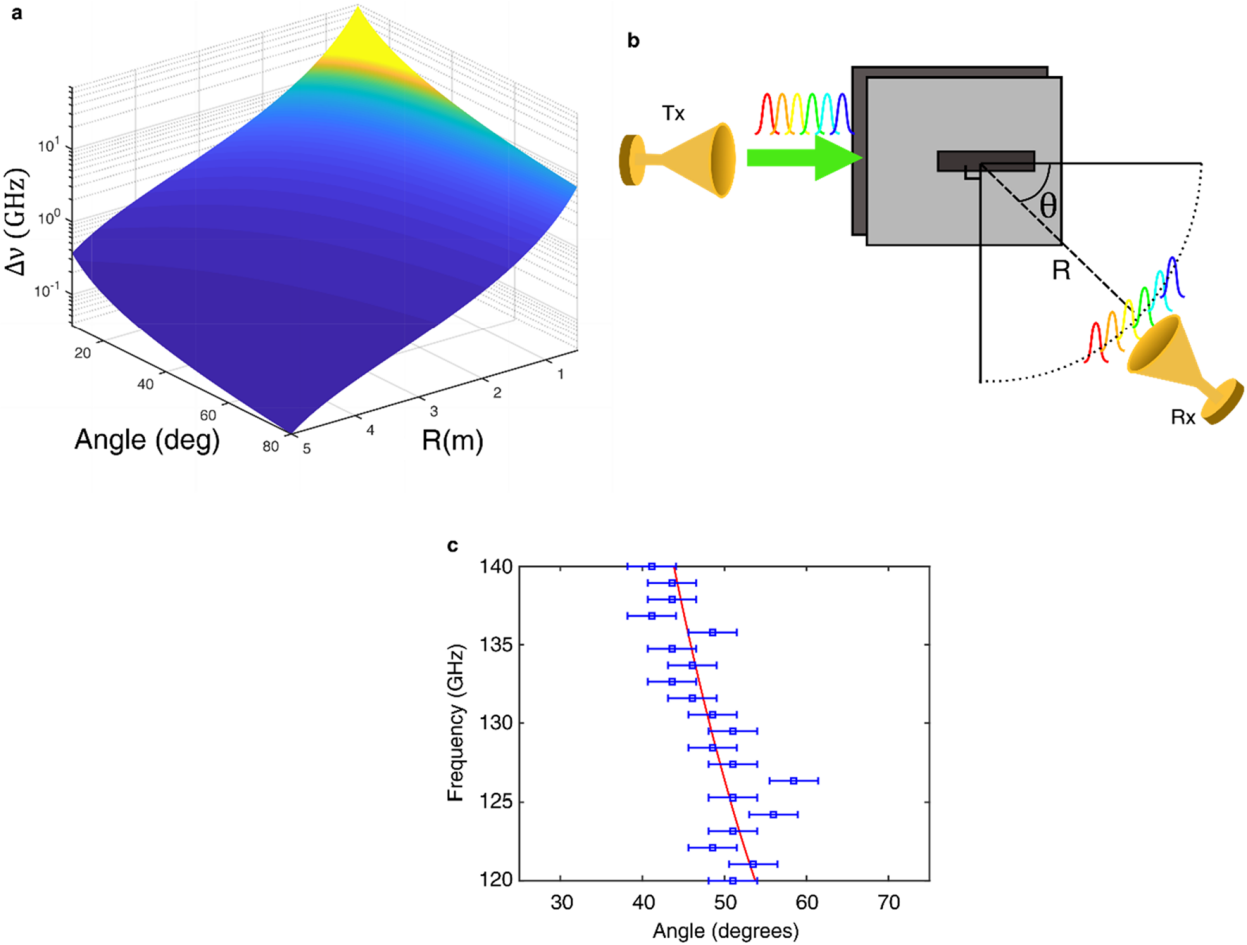
(**a**) The calculated spectral bandwidth, $\mathrm{\Delta \nu }$, of the system decreases nonlinearly as the angle and distance is increased. (**b**) Experimental setup for measuring the radiated frequency components from the leaky wave antenna. The LWA transmits 20 tones from 120 to 140 GHz (1 GHz separation) and the signal power is detected at a receiver with a radial distance of $\mathrm{R}$ and angle $\uptheta$. (**c**) The angular location of the peak detected power of each tone between 25° and 75°, at a distance of 200 cm. The maximum power of each tone shifts from 45 to 53 degrees. The error bars indicate the 3-degree uncertainty in angular position. This shift in peak matches the analytical calculations from Eq. (4) (red line).

Likewise, the received bandwidth is the same as Eq. (3) with the grating period Λ replaced by 2*b*. We make the same approximation for the acceptance angle, $\Delta \theta \approx \frac{{A}_{apt}}{R}$, for a given aperture ${A}_{apt}$ and transmit distance $R$. Similar to the diffraction grating, if a broadband signal is transmitted, the angular dispersion of the leaky wave antenna spreads the beam in space and the receiver is able to only detect a portion of the signal spectrum. The received spectral bandwidth, $\Delta \nu$, is calculated for angles from 10° to 80° and distances from 0.5 to 5 m and shown in Fig. 3a, under the assumption that the transmitted bandwidth is large enough so that the angular aperture of the receiver is the limiting factor. This plot displays the strong and nonlinear decrease in bandwidth with angle and distance from the transmitter. Clearly, this variation in received bandwidth can have an important impact on the achievable data rate in broadband transmissions.

In order to model the filtering effect from the detector aperture on the received signal, we calculate the FSPL, the transmitted radiation pattern of the LWA, and the filtering aperture of the receiving antenna. The product of these factors (Eq. 5) provides a prediction of the power of the received signal at a given angle,${\theta }_{Rx}$, wavelength,$\lambda$, and receiver distance $R$. The radiation pattern of the LWA, $S\left(\lambda ,\theta \right),$ is determined by the far-field diffraction pattern of the slot aperture^28^

5 $$S\left(\lambda ,\theta \right)={\left(Lsinc\left[\left(\beta -{k}_{0}\mathrm{sin}\left(\theta \right)-j\alpha \right)\left(\frac{L}{2}\right)\right]\right)}^{2}$$

Here $\beta = \sqrt{{k}_{0}^{2}-{\left(\frac{\pi }{b}\right)}^{2}}$ is the wavevector of the TE_1_ mode, ${k}_{0}$ is the free space wavevector, $L$ is the slot length, and $\alpha$ is the attenuation of the TE_1_ mode due to radiation loss. For this factor, we use an empirically determined value of 28 m^−1^ ^26^. The filtering aperture function, $G({\theta }_{Rx},\Delta \theta )$, describes the angular aperture of the receiver. We model this as a Gaussian centered at angle ${\theta }_{Rx}$ with a width determined by the acceptance angle $\Delta \theta$ inversely proportional to $R$. To account for the angular dependence of both this aperture and the transmitted signal’s radiation pattern, we integrate the product of these two functions over all angles (Eq. 6), which constitutes a prediction of the received power given by

6 $$\upgamma \left(\lambda ,{\theta }_{Rx},\Delta \theta \right)={\left(\frac{c}{4\pi fR}\right)}^{2}{\left({\int }_{0}^{2\pi }\left[{\left(Lsinc\left[\left(\beta -{k}_{0}\mathrm{sin}\left(\theta \right)-j\alpha \right)\left(\frac{L}{2}\right)\right]\right)}^{2}\mathrm{exp}\left(-\frac{{\left(\theta -{\theta }_{Rx}\right)}^{2}}{2\Delta {\theta }^{2}}\right)\right]d\theta \right)}^{2}$$

An illustration of the experimental setup is shown in Fig. 3b. In our experiments, we generate a signal at a series of frequencies in the range 120–140 GHz signal, at 1 GHz intervals, and measure the attenuation of each of these spectral components at several different angles and transmit distances. These signals are generated by a custom VDI amplifier frequency multiplier chain. An integrated mixer is utilized to modulate the carrier signal with the information-bearing baseband signal. At the receiver, a low noise mixer is used to obtain the IF signal from RF. The transmitter and receiver mixers are driven by a local oscillator at a frequency of 30 GHz and 35 GHz. The baseband signal is generated and processed using an offline digital back-end system based on an arbitrary waveform generator at the transmitter. We use a digital storage oscilloscope at the receiver to capture the data^29–31^. The LWA is butt-coupled to the VDI rectangular waveguide such that the rectangular TE_10_ mode is coupled into the TE_1_ mode inside the LWA with an estimated efficiency of 70%. Our receiver is a horn antenna with a diameter of 1.07 cm and gain of 21dBi. Initially, we place the receiver at a distance of 200 cm, and measure the received power of each tone at angles from 25° to 75°. As expected, the measured tones from 120 to 140 GHz produce radiation patterns whose peaks shift in angle according to Eq. (4) (see Fig. 3c). The experimental results show a good match to the analytical frequency-angle relation (red line).

**Figure 4:**
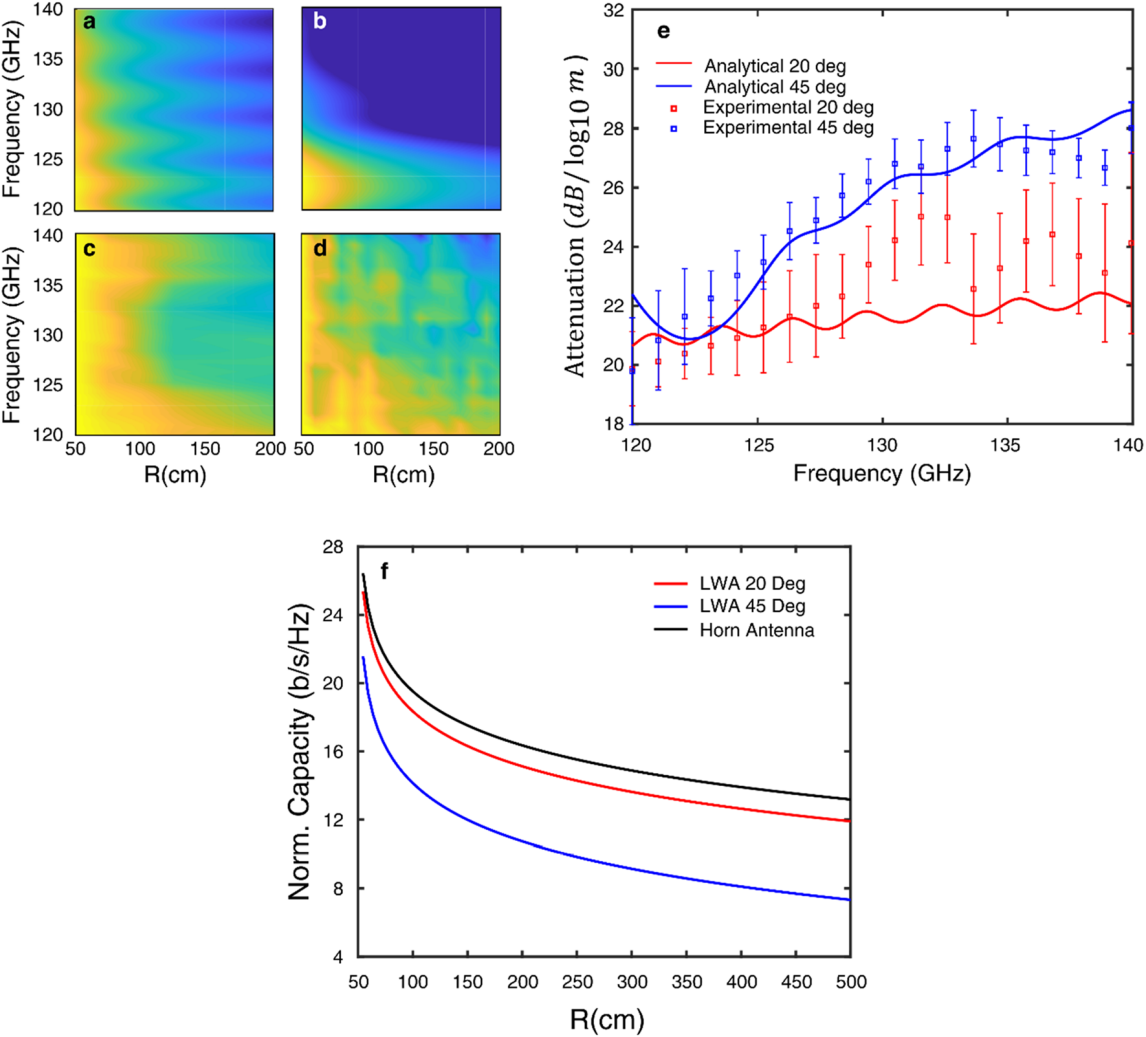
The receiver is positioned at $\uptheta =20^\circ , 42^\circ$ and the distance, $\mathrm{R}$ is increased from 0.5 to 2 m . (**a**) The results of analytical model from Eq. (5) at ${\uptheta }_{0}=20$° and (b) at ${\uptheta }_{0}=45$°. The experimentally measured power of each of the 20 tones plot as a function of distance at (**c**) $20$° and (**d**) $45$°. The attenuation, $\mathrm{A },$ is extracted from fitting the results to Eq. (7). (**e**) The attenuation calculated from the analytical and experimental results show an increase in attenuation at $45$° compared to $20$°, and this increases in frequency. The error bars show the 95% confidence interval for each of the fit. The average sum of squared residuals was 0.755 and 0.879 for the $45$° and $20$° fits. (**f**) The calculated Shannon capacity for a 122.5 GHz signal with a bandwidth of 5 GHz, as a function of distance for a link using a LWA or a traditional horn antenna (black line). The horn antenna link experiences FSPL, whereas a LWA transmitting to a receiver positioned at 45° (blue line) undergoes increased path loss resulting in a lower capacity. Comparatively, if the receiver were to be positioned 20° (red line), the path loss is less and the capacity is greater.

Next we explore the effect of the decreasing spectral bandwidth on the signal attenuation. We position our receiver at two different angles (20° and 45°), and measure the attenuation of the signal vs. transmit distance. We compare these results to the analytical expression (Eq. 6). The radiation pattern of the analytical calculation, shown in Fig. 4a,b, match the measured experimental results shown in Fig. 4 c-d. In order to characterize the increase in attenuation at the two angles, we fit the normalized power of each tone to the following expression as a function of frequency, and distance.

7 $$PL= Alo{g}_{10}\left(\frac{c}{4\pi R{\nu }_{tone}}\right)$$

Here ${\nu }_{tone}$ is the frequency of the given tone, and $A$ is the attenuation of the signal (dB/decade). An isotropic antenna would radiate each tone such that the beam experiences FSPL of 20 dB/decade, so the $A$ parameter would be equal to 20. Fig. 4e shows the results of fitting the calculation and experimental data to Eq. (7). The plot reveals several important results. First, the fitting parameter A, attenuation per decade, is greater than that of simple free-space path loss (20 dB per decade), due to the variation in spectral acceptance bandwidth with distance. Second, we find that the rate of increase in the attenuation is different for different frequencies, with greater loss at 45° compared to 20°. This can be understood as the result of the nonlinear variation of the change in the spectral bandwidth with angle and distance. In essence, the system becomes bandwidth limited because the receiver acts as a filter and is unable to detect the majority of the original bandwidth, due to the angular spreading of spectral components.

To illustrate the impact of this behavior, we compute the Shannon capacity, given by

8 $$C=Blo{g}_{2}\left(1+SNR\right)$$

using the attenuation from Fig. 4e. Here, $B$ is the bandwidth and the SNR is calculated using the received signal power, according to

9 $$SNR={P}_{Tx}+{G}_{Tx}+{G}_{Rx}-PL-{N}_{0}$$

For this calculation, we envision a communication link using a LWA transmitting a 122.5 GHz signal, with 5 GHz of bandwidth, and 12dBm of transmitted power, $${P}_{Tx}$$. A receiver is positioned at either 45° or 20° and a given distance from the transmitter. We assume both the transmitter and receiver have a gain of 21 dBi ($${G}_{Tx}={G}_{Rx})$$ and experience −65 dBm of thermal noise, $${N}_{0}$$. The path loss ($$PL$$) is calculated using

10 $$PL=\frac{1}{N}\sum_{i}^{N}\left({A}_{i}{\mathrm{log}}_{10}\left(R\right)+{A}_{i}{\mathrm{log}}_{10}\left({f}_{i}\right)-147.55\right)$$

where this quantity is averaged over the number of tones, $N$. In Eq. (10), $R$ is the distance in meters, ${A}_{i}$ is the attenuation taken from Fig. 4e for each frequency tone, ${f}_{i}$ (given in Hz), and the 147.55 term is derived from the log of the constants in FSPL, $10lo{g}_{10}\left({\left(\frac{4\pi }{c}\right)}^{2}\right)$. The resulting Shannon capacity calculation, presented in Fig. 4f, shows a lower capacity at a receiver position of 45° compared to 20°. For comparison, a link with a transmitting horn antenna with identical antenna gain, transmit power and noise experiencing only FSPL is also presented. The LWA has a decreased capacity due to the spread of the bandwidth and filtering effect from the receiver compared to a horn antenna.

**Figure 5:**
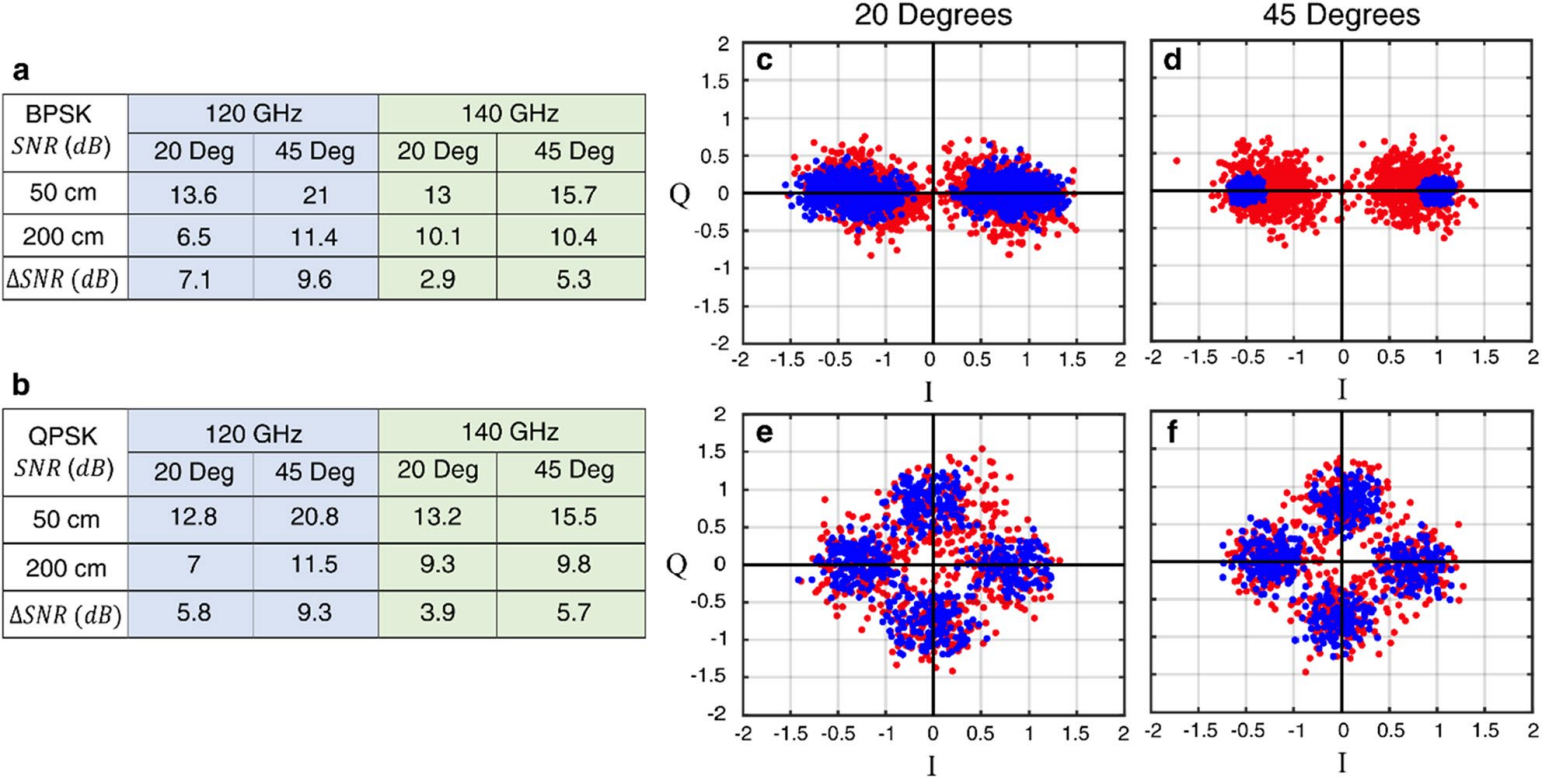
(**a**) A LWA is used to transmit a 2.5 giga samples per second (Gsps) BPSK signal centered at 120 GHz and 140 GHz. The receiver is positioned at 20° and 45° and moved from 50 and 200 cm. (**b**) The same measurement is repeated using QPSK modulation. The higher attenuation at 45°, compared to 20°, results in a greater SNR loss. This result is seen for both carrier frequencies and both modulation schemes. The SNR flutuated by 0.6 and 0.3 dB for the 120 GHz and 140 GHz system respectively. (**c,d**) The constellation diagram for a 140 Ghz signal carrying 2.5 Gsps BPSK signal at 20° and 45°. (**e,f**) The same measurement repeated for QPSK at 20° and 45°. The blue dots are data points measured at 50 cm and the red dots are points at 200 cm. The BPSK illustrate the greater increase in noise at 45° compared to 20°.

Finally, we show the effect of angular dispersion on a communication link by transmitting a 2.5 Gbaud/s binary phase shift keyed (BPSK) and a quadrature phase shift keyed (QPSK) signal. We transmit each signal through the LWA at carrier frequencies of 120 GHz and 140 GHz. The receiver is positioned at angles of 20° and 45° and distances of 50 cm and 200 cm. The attenuation in SNR (dB) resulting from moving the receiver from 50 to 200 cm is tabulated in Fig. 5a,b. Clearly, the decrease in SNR is greater at 45° than at 20° for both modulation schemes. This result is consistent with the increased attenuation at higher angles, as noted above. The increase in attenuation at 45° leads to a faster deterioration of the signal, resulting in a lower SNR. The constellation diagrams of the received BPSK and QPSK (Fig. 5c–f) illustrates the increase in noise at 45° compared to 20°. As the bandwidth of the data, and the receiver distance increases the impact on signal quality is also expected to be more severe. In modulation schemes sensitive to the bandwidth, this effect will be more pertinent.

## Discussion and conclusion

We have shown the frequency-angle dependent attenuation resulting from the angular dispersion a LWA and a diffraction grating. The nonlinear frequency-angle property that allows for beam-steering a signal, also gives rises to angular dispersion which ultimately reduces the spectral bandwidth of the link. Angular dispersion, if left uncorrected, spreads the frequency components in space and make higher frequency modulation components unavailable for detection. This signal loss resulting from this effect exceeds the regular 20 dB/decade commonly experienced in FSPL. In the case of the LWA being used as the transmitter, the capacity will be limited based on the receiver position and distance and the increased loss is unavoidable for the receiver but can potentially be mitigated by external optics or alternative slot design^26^. Alternatively, spectral efficiency protocols can be implemented to mitigate the effect of the loss in capacity at given receiver positions and distances.

In the RF regime, the effect of diffractive elements in a link have been thoroughly studied and well documented^32^. Angular dispersion in a communication setting has been considered in the ultra-wideband (UWB) regime and in 5G systems^33–36^. However, as frequencies increase to the THz regime and beyond, the effects of angular dispersion will become even more significant^37,38^. If multiple angularly dispersive devices are placed in a channel, the affect will accumulate and could severely limit the spectral bandwidth at the receiver. In the case of effective beam-steering, the potential of using diffractive elements is an ongoing investigation^39,40^. The impact of such devices on the spectral bandwidth and any additional loss introduced must also be thoroughly considered. This can be extended to other diffractive devices that also introduce similar dispersion. For example, a reflective textured wall, an aperture, or a metasurface designed to reflect THz waves at specific directions^41^ can also introduce angular dispersion through the frequency-dependence of diffraction patterns. Such effects will need to be carefully considered in future system design.

## Data Availability

The authors declare that the data generated or analyzed during this study are included in this article.
